# Automatic seizure detection based on imaged-EEG signals through fully convolutional networks

**DOI:** 10.1038/s41598-020-78784-3

**Published:** 2020-12-11

**Authors:** Catalina Gómez, Pablo Arbeláez, Miguel Navarrete, Catalina Alvarado-Rojas, Michel Le Van Quyen, Mario Valderrama

**Affiliations:** 1 Department of Biomedical Engineering, Universidad de los Andes, Bogotá, Colombia; 2 Center for Research and Formation in Artificial Intelligence (CINFONIA), Universidad de los Andes, Bogotá, Colombia; 3 School of Psychology, Brain Research Imaging Centre, Cardiff University, Cardiff, UK; 4 Department of Electronic Engineering, Pontificia Universidad Javeriana, Bogotá, Colombia; 5 Laboratoire d’Imagerie Biomédicale (LIB), Inserm U1146 / Sorbonne Université UMCR2 / UMR7371 CNRS, 15 rue de l’Ecole de Médecine, 75006 Paris, France

**Keywords:** Learning algorithms, Electroencephalography - EEG, Epilepsy

## Abstract

Seizure detection is a routine process in epilepsy units requiring manual intervention of well-trained specialists. This process could be extensive, inefficient and time-consuming, especially for long term recordings. We proposed an automatic method to detect epileptic seizures using an imaged-EEG representation of brain signals. To accomplish this, we analyzed EEG signals from two different datasets: the CHB-MIT Scalp EEG database and the EPILEPSIAE project that includes scalp and intracranial recordings. We used fully convolutional neural networks to automatically detect seizures. For our best model, we reached average accuracy and specificity values of 99.3% and 99.6%, respectively, for the CHB-MIT dataset, and corresponding values of 98.0% and 98.3% for the EPILEPSIAE patients. For these patients, the inclusion of intracranial electrodes together with scalp ones increased the average accuracy and specificity values to 99.6% and 58.3%, respectively. Regarding the other metrics, our best model reached average precision of 62.7%, recall of 58.3%, F-measure of 59.0% and AP of 54.5% on the CHB-MIT recordings, and comparatively lowers performances for the EPILEPSIAE dataset. For both databases, the number of false alarms per hour reached values less than 0.5/h for 92% of the CHB-MIT patients and less than 1.0/h for 80% of the EPILEPSIAE patients. Compared to recent studies, our lightweight approach does not need any estimation of pre-selected features and demonstrates high performances with promising possibilities for the introduction of such automatic methods in the clinical practice.

## Introduction

Epilepsy is a chronic neurological condition characterized by an enduring propensity to generate epileptic seizures, which are transient aberrations in the brain’s electrical activity. Epileptic patients often suffer from several neurobiological, cognitive, psychological and social impairments. This disease affects over 50 million people worldwide^1^. EEG recordings of patients suffering from epilepsy show two types of activities: interictal (time span between seizures) and ictal (time span between seizure onset and offset).

In the clinical practice, diagnosing epilepsy requires a visual analysis of electroencepaholographic (EEG) recordings performed by experienced neurophysiologists, who identify characteristic patterns of the interictal and ictal activities^2^. Due to the spontaneous nature of epileptic seizures, continuous and long-term EEG recordings of patients are needed (with durations ranging from hours to days) since their main purpose is to record seizures that occur without warning. This is particularly true for patients implanted with intracranial electrodes as part of an invasive exploration leading to the removal of the epileptic tissue, for whom recordings can last several days or even weeks in a hospital room under continuous monitoring. Due to this, resulting EEG signals could be extremely extensive, making their analysis a very costly, inefficient and time-consuming process that requires the interpretation of well-trained specialists. In addition, visual inspection of EEG data could lead to disagreements among specialists on the same recording due to the subjective analysis. Similarly, EEG patterns that characterize a seizure can be missed or confused with background noise or artifacts, and there is cross-patient variability in seizure and non-seizure activity. Furthermore, the access to a neurologist could be unavailable or very reduced in developing countries^3^.

The drawbacks of manual inspection call for methods that implement automatic seizure detection emulating visual analyses performed by experts. In a clinical setting, the acceptance of these algorithms depends on a low false-positive detection rate, and a high sensitivity. The characteristic frequency bands of the brain activity have traditionally called for frequency analysis of EEG recordings. Traditional methods change signals to the frequency domain by using the Fourier or wavelet transforms, to extract relevant features that describe a seizure, and often combine them with time-domain descriptors. These hand-crafted features are then the inputs to train a classifier capable of distinguishing between seizure and non-seizure episodes (for complete recent reviews refer to^4,5^).

The limitations of using domain-based methods is that they are susceptible to variations in seizure patterns because of the acquisition artifacts and the non-stationary nature of the EEG, which makes its statistical components to change across subjects and time^6^.

Deep learning has been widely used for automated EEG processing in different contexts such as brain computer interfaces^7–10^, automatic sleep scoring^11–14^ and epileptic seizure prediction^15–17^ and detection^18–23^, due to its capacity to learn rich representations from raw data^24^ and its successful performance on visual recognition tasks, specially on natural images^25^. In particular, for the automatic seizure detection problem, recent approaches are summarized in Table 1. In this table, the first two columns refer to the method, the third to the database used for the evaluation, and the last ones to the reported metrics, when available, for evaluating the algorithm: classification and sensitivity in the range [0, 100], latency in seconds and false positive rate per hour (FPR/h). In the following paragraphs we describe more in detail some of these studies.

Recent contributions to the application of deep learning methods for seizure detection have been developed on the dataset provided by the University of Bonn^26^. This database allows the study of a variety of tasks since it comprises intracranial EEG signals of interictal and ictal episodes from epileptic patients, as well as normal scalp recordings of healthy subjects. In particular, in^23^, a single Recurrent Neural Network (RNN) was proposed to exploit the temporal dependencies in EEG signals, which was evaluated in a binary (normal EEG vs. ictal) and a multi-class classification (normal vs. interictal vs. ictal) schemes. In^27^, a 13-layer Convolutional Neural Network (CNN) was evaluated on a variant of the multi-class problem, which directly included the pre-ictal category. In the binary task, the authors of^21^ defined an augmentation scheme with overlapping windows to alleviate the few available data to train deep learning models. In addition, they introduced a pyramidal one-dimensional CNN that leverages local information to generate a final prediction. Although the previous methods successfully detected seizures segments, they require additional adjustments to process multi-channel data, and more importantly, they need to be validated on long-term EEG signals since the very short duration of recordings in this database (23.6s) prevents an evaluation of their robustness and generalization capabilities.

In parallel to the Bonn dataset, several other studies tested their algorithms for seizure detection using the CHB-MIT Scalp EEG database^3,28^. The advantage of this collection of signals is that it contains continuous long-term recordings that can last several hours, which, combined with detailed clinical annotations about seizures times, makes it very suitable for comparisons between different methods. In particular, in^29^, the authors proposed a deep learning approach in a supervised learning framework to automatically learn more robust features. They used a RNN architecture in a leave-one-out scheme that captures spectral, spatial and temporal patterns representing a seizure. In addition, a novel input representation was proposed in which an image representation of EEG signals was created combining spatial information (electrodes montage) with the magnitude of different frequency bands as the pixel attribute. In^20^, a CNN model based on multilayer perceptrons was proposed in which time domain signals were transformed to frequency domain features by calculating the power spectrum density for 10-s windows. In addition to Sensitivity, they report more reliable metrics, such as Precision and F-measure, obtaining a performance of around 95% for this last one. Nevertheless, the authors chose only 8 h of known non-seizure data for each patient, which does not correspond to the actual class imbalance present in the database. In a similar approach^30^, EEG signals were transformed to power spectrum density energy diagrams which are fed into a deep CNN in a transfer learning strategy to classify four categories of epileptic states. Using a subset of 11 patients and only 116 min for the inter-ictal and pre-ictal classes from this dataset, they obtained an average classification accuracy of 90% for seizure episodes. Alternatively, using raw EEG signals in the time domain, the authors in^31^ trained a CNN model to detect inter-ictal epileptic discharges as well as seizure onsets. They obtained an overall seizure detection sensitivity of around 90% but, as in other studies, only a subsample of all available data limited to few hours was used to train and evaluate their model. In another study^32^, the authors employed a CNN to compare the seizure detection performance from time and frequency domains through the use of raw signals and their corresponding spectral representation respectively. In addition to scalp EEGs, the algorithms were also tested in intracranial recordings from a different dataset. Surprisingly, while they obtained high performances for the frequency domain scheme, the metrics for the time domain strategy in scalp recordings were very low compared to other approaches (see Table 1).

Using different datasets, other approaches have evaluated alternative input modalities for CNNs in seizure detection. For instance, in^18^, in addition to 5-s raw time-series as input, the authors explored a transformed black and white image of the raw waveforms, a periodogram between 0 and 99 Hz, and a gray scale image of the short-time Fourier transform. These data representations were extracted from intracranial EEGs (single channel) from a mouse model of epilepsy, and further validated on a human intracranial EEG dataset. For each input modality, different architectures were evaluated depending on the dimensionality of the data (1D or 2D). In another study, the authors proposed the use of 3D inputs from multi-channel EEG recordings^22^; the results over 13 patients with 159 seizures demonstrated that 3D models surpassed 2D models in metrics such as accuracy, specificity and sensitivity in about two points. However, 3D models have a larger number of parameters to be learned, and thus require much more training samples. An alternative to reduce the number of parameters was proposed in^19^ using Fully Convolutional Networks (FCN) for neonatal seizure detection. The FCN architecture had a Global Average Pooling layers that replaced the fully connected ones. The experiments were conducted on a large dataset of continuous multi-channel neonatal EEG totaling 834 h and 1389 seizures from 18 newborns, and tested on a public dataset of neonatal EEG waveforms collected at Helsinki University Hospital. The area under the receiver-operating curve (AUC) for specificities over 90% was 86.9% for the 18 test patients.

In this work, we address the seizure detection problem following a deep learning strategy derived from robust methods for object recognition tasks in the computer vision field. Our intention is to mimic the visual inspection performed by clinical specialists when reading EEG recordings. For this, we propose a strategy to generate image representations of raw EEG signals that we use as inputs to our seizure detection algorithms. We tested our strategies in two different datasets, one with only scalp electrodes and the other with combined scalp and intracranial recordings. Our lightweight model with only 314 thousand parameters and minimal pre-processing steps reached high seizure detection performance, comparable or even improving previous studies in which signal transformations are needed for its implementation. Furthermore, in addition to the evaluation on a standard database for seizure detection, we had the special opportunity to compared our seizure detection algorithm on scalp and intracranial recordings in the same subject and to evaluate transfer learning strategies between these modalities, as we have simultaneous signals from the EPILEPSIAE dataset.

**Table 1:** Performance comparison of recent seizure detection studies with automated models.

Method	Model	Database	Sensitivity	Specificity	Latency (s)	FPR/h
Acharya et al.^27^	CNN	University of Bonn	95	90	–	–
Ullah et al.^21^	CNN	University of Bonn	98	98	–	–
Hussein et al.^23^	RNN	University of Bonn	100	100	–	–
Shoeb^3^	SVM	CHB-MIT Scalp EEG	96	–	4.6	0.08
Thodoroff et al.^29^	RNN	CHB-MIT Scalp EEG	85	–	–	0.8
Birjandtalab et al.^20^	CNN	CHB-MIT Scalp EEG	96.3	–	–	–
Zhou et al.^32^	CNN	CHB-MIT Scalp EEG	61	63	–	–
Cho et al.^18^	2D CNN	iEEG mouse model	96.9	99.9	–	–
Wei et al.^22^	3D CNN	Hospital of Xinjiang Medical University	88.6	93.8	–	–

## Results

### CHB-MIT Scalp EEG

**Figure 1:**
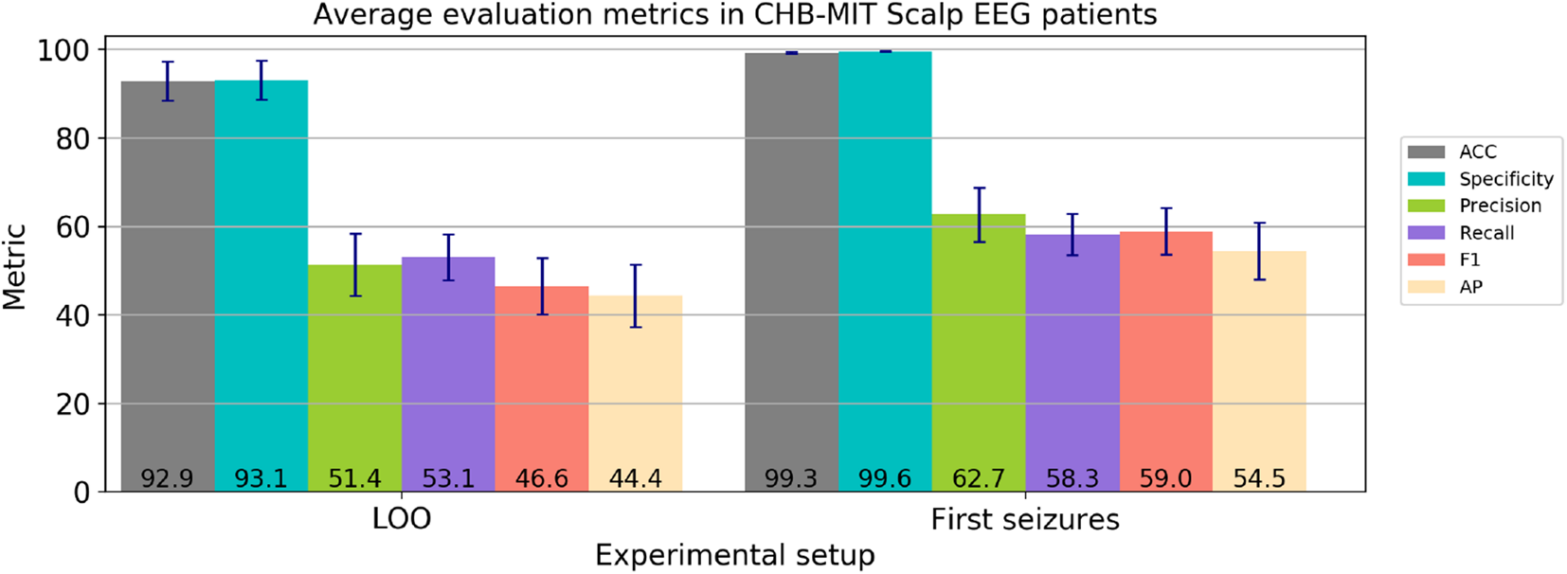
Average metrics for the LOO and First Seizures models evaluated in the CHB-MIT Scalp EEG database. Each color bar corresponds to a different metric, with the average value at the bottom and the standard error of the mean ($\sigma / \sqrt{N}$) as blue lines.

In order to choose the best architecture for our detection model, we explored several configurations of Fully Convolutional Networks (FCN) in the 3-Fold Cross Validation (3FCV) setup (see the Methods section and Table 2 in the supplementary material), and with the best configuration, we trained and evaluated the Leave-One-Patient-Out (LOO) model to evaluate the model’s capacity to detect seizures on data from unseen patients. The best model configuration is a FCN with 3 blocks, 128 filters, and dropout layers with probabilities of 0.1 and 0.3 at the early and last convolutional layers, respectively. We included a *L2* regularization (weight decay) that helps the network to generalize better. The results are summarized in Fig. 1 (left bar graphs). For all patients, we obtained global accuracy and specificity levels of $92.9\pm 21.8$ (MEAN ± STD) and $93.1 \pm 21.9$, respectively. In addition, we reached global values for precision of $51.4\pm 34.1$, recall of $53.1\pm 25.5$, F-measure of $46.6\pm 31.0$ and the AP $44.4\pm 34.4$.

**Figure 2:**
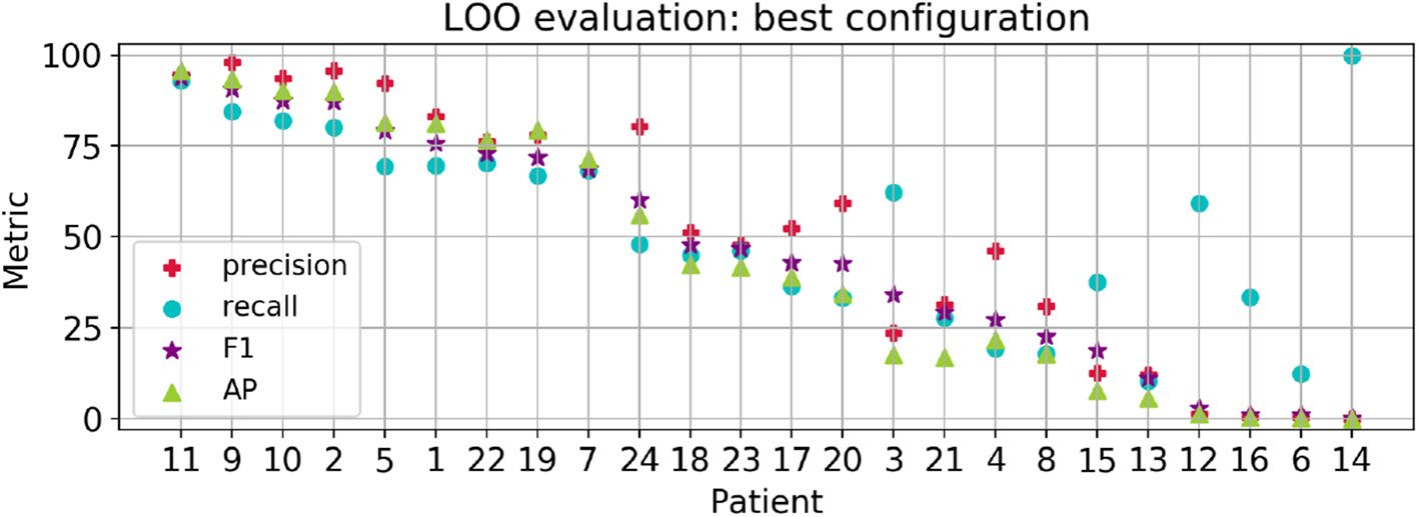
Results for the LOO model with the best configuration. Patients in the x-axis are sorted by decreasing F-measure (purple star marker).

**Figure 3:**
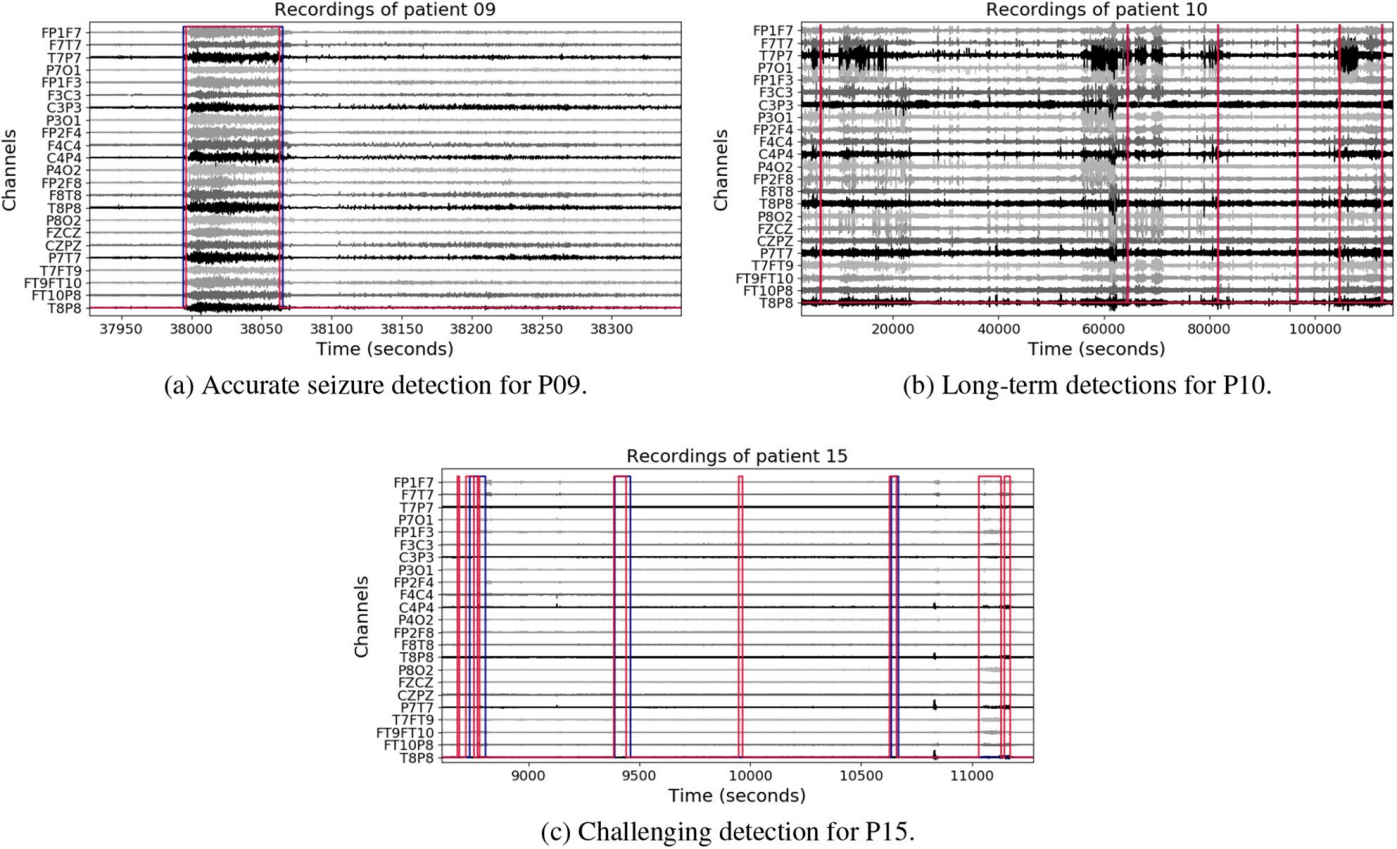
Qualitative results for the best LOO model (base FCN + DO). Annotations (blue) and predictions (red) are plotted over the signals. The bipolar configuration of electrodes is shown in the y-axis.

Individual metrics per patient are presented in Fig. 2. Overall, 33% of the patients reached a F-measure greater than 70% (n = 255 recordings), 20% obtained a precision over 90% (n = 153 recordings) and another 20% a recall over 80% (n = 140 recordings). When we contrasted the performance with the number of seizures per patient, we noticed that the metrics tended to decrease with an increasing number of seizures, whereas patients with less seizures have both high precision and recall values (see for instance patients 11, 9 and 2). Qualitative results of accurate detections and limitations are shown in Fig. 3. In this figure, we provide an example of an accurate match between the annotated and detected seizure times (Fig. 3A); an example of long-term accurate detections in more than 25 h of recording (the ground truth lines are covered by the detections in red, Fig. 3B), and a segment with false positives and disrupted detections (Fig. 3C).

**Figure 4:**
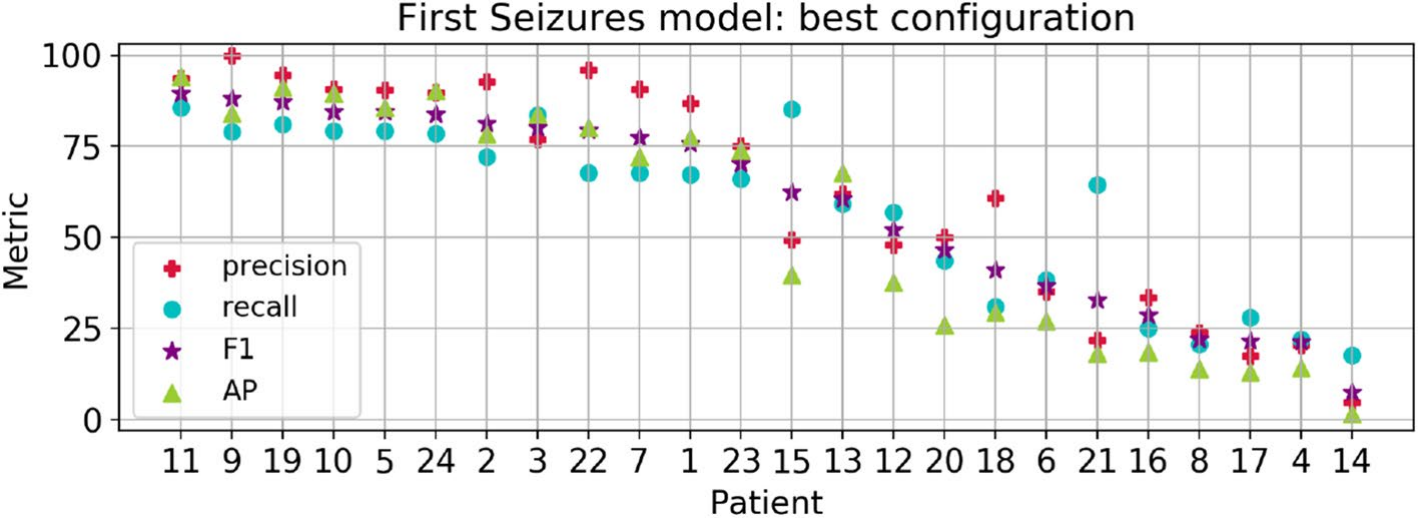
Results of the First Seizures model for all patients. Patients in x-axis are sorted by decreasing F-measure.

Based on the results obtained individually, we identified some patients as difficult, with seizures harder to detect than others. Thus, in order to exploit the model’s capacity to learn from hard seizure instances, and therefore to increase the performance for challenging patients, we proposed to train another cross-patient model with data from the first 80% of recordings with seizures and evaluate in the remaining ones, referred as First Seizures model in Fig. 1. Compared to the performance in the best LOO model, the average accuracy and specificity were notably higher for First Seizures setup, reaching values of $99.3\pm 1.16$ and $99.6\pm 0.59$, respectively. In addition, the precision ($62.7\pm 30.2$), F-measure ($59.0\pm 25.9$) and AP ($54.5\pm 31.5$) for the First Seizures strategy improved in more than 10 points while the recall increased by 5 points ($58.3\pm 22.9$). Furthermore, we obtained a noticeable improvement for patients with seizures hard to detect. For instance, for patient 12 the precision increased from 1.55 (in the LOO model) to 48.1, while keeping almost the same recall (60%). For each patient, the performance in the First Seizures model is presented in Fig. 4. For a comparison of the performance of individual patients under the three different cross-patient models see Table 3 in the supplementary material.

**Figure 5:**
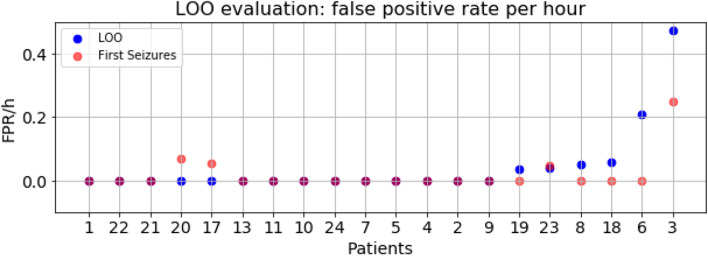
False positive rate per hour defined in consecutive 10s windows for the best LOO model (blue) and First Seizures model (red). Patients are sorted by increasing FPR/h. The y-axis is cut at FPR/h value of 0.5 to visualize minor differences. Patients 15, 16, 12 and 14 have values over 2 and are not shown in the plot.

For the LOO and First Seizures models, we calculated the false positive rate (FPR) as in^33^. In particular, we defined the false alarm rate as the number of false alarms per hour of recording. To count the false alarms in the predictions, we analyzed consecutive 30-s segments of signal, as defined in^29^. First, we changed from class probabilities to binary predictions using a threshold corresponding to the best F-measure, and then, following the standard evaluation for object detection^34^, if more than the 50% of the window was predicted as the seizure class, the window was compared to the corresponding time segment in the annotations. Figure 5 presents the FPR/h for all patients individually and for both model configurations. In the LOO model, the average FPR/h was $7.80 \pm 26.1$ ranging from 0 to 119.9. Even though the false alarm rate was distributed within a wide range, 83% of the patients obtained a rate below 0.5 false alarms per hour, with an average FPR/h for the values within the 95% percentile equal to $0.39\pm 1.15$. The FPR/h range for all patients decreased considerably in the First Seizures model, [0–2.14], with an average of $0.14 \pm 0.45$.

### EPILEPSIAE

**Figure 6:**
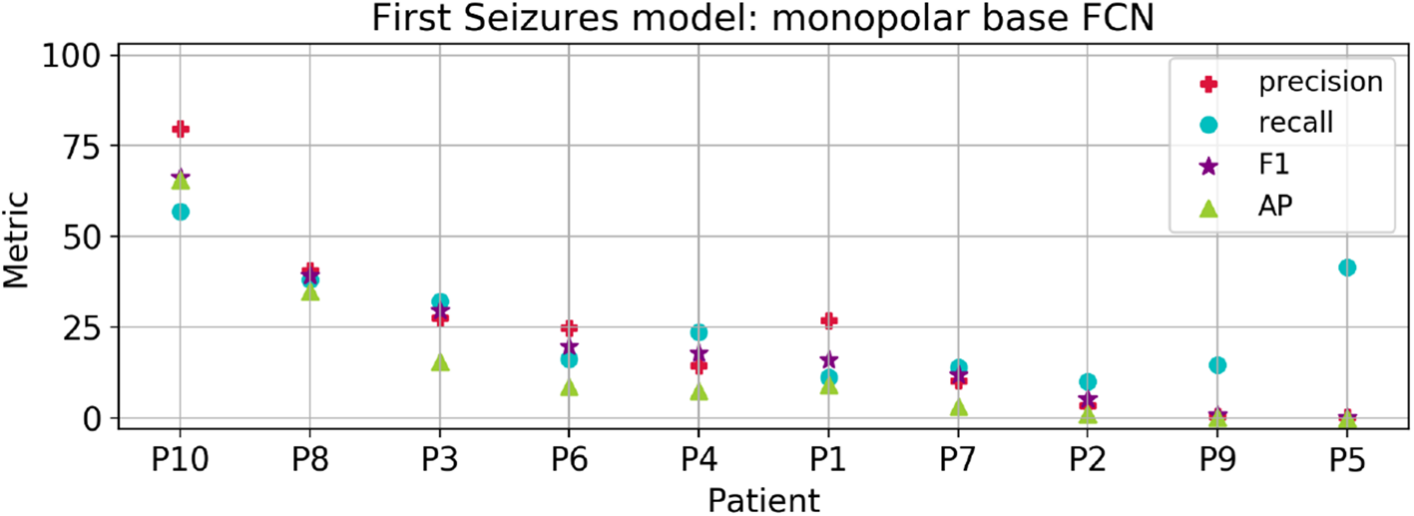
Average metrics for the best experiment in EPILEPSIAE patients using scalp electrodes only. Patients in the x-axis are sorted by decreasing F-measure (purple star marker).

To compare the results between the EPILEPSIAE and CHB-MIT Scalp datasets, we first carried out experiments using only scalp electrodes from the former database in the First Seizures setup. We departed from the parameter exploration conducted in the CHB-MIT dataset and used the base FCN with Dropout (DO) and weight decay. For this model configuration, we explored a finetuning strategy using the First Seizures model in CHB-MIT patients as the weight initialization. Besides, we evaluated models trained from scratch with electrodes in monopolar and bipolar configuration. For a complete comparison of the experiments in EPILEPSIAE patients with scalp recordings refer to Fig. 1 in the Supplementary Material. We summarize the best results achieved with the monopolar model in Fig. 6. Among all patients, we obtained an average F-measure of $20.8 \pm 19.1$, an AP of $14.7\pm 19.6$, a recall of $25.9 \pm 14.9$ and a precision of $22.9\pm 22.8$. Overall, these metrics showed a strong variation among patients, and a low performance for 8 out of 10, which is reflected in the reduced average performance. However, the average accuracy of $98.0\pm 3.34$ and specificity of $98.3\pm 3.4$ are not affected because of the large amount of true negatives. In addition, we calculated the number of false alarms per hour obtaining an average among the 10 patients of $1.20 \pm 2.66$ (ranging from 0.0 to 9.08 FPR/h).

**Figure 7:**
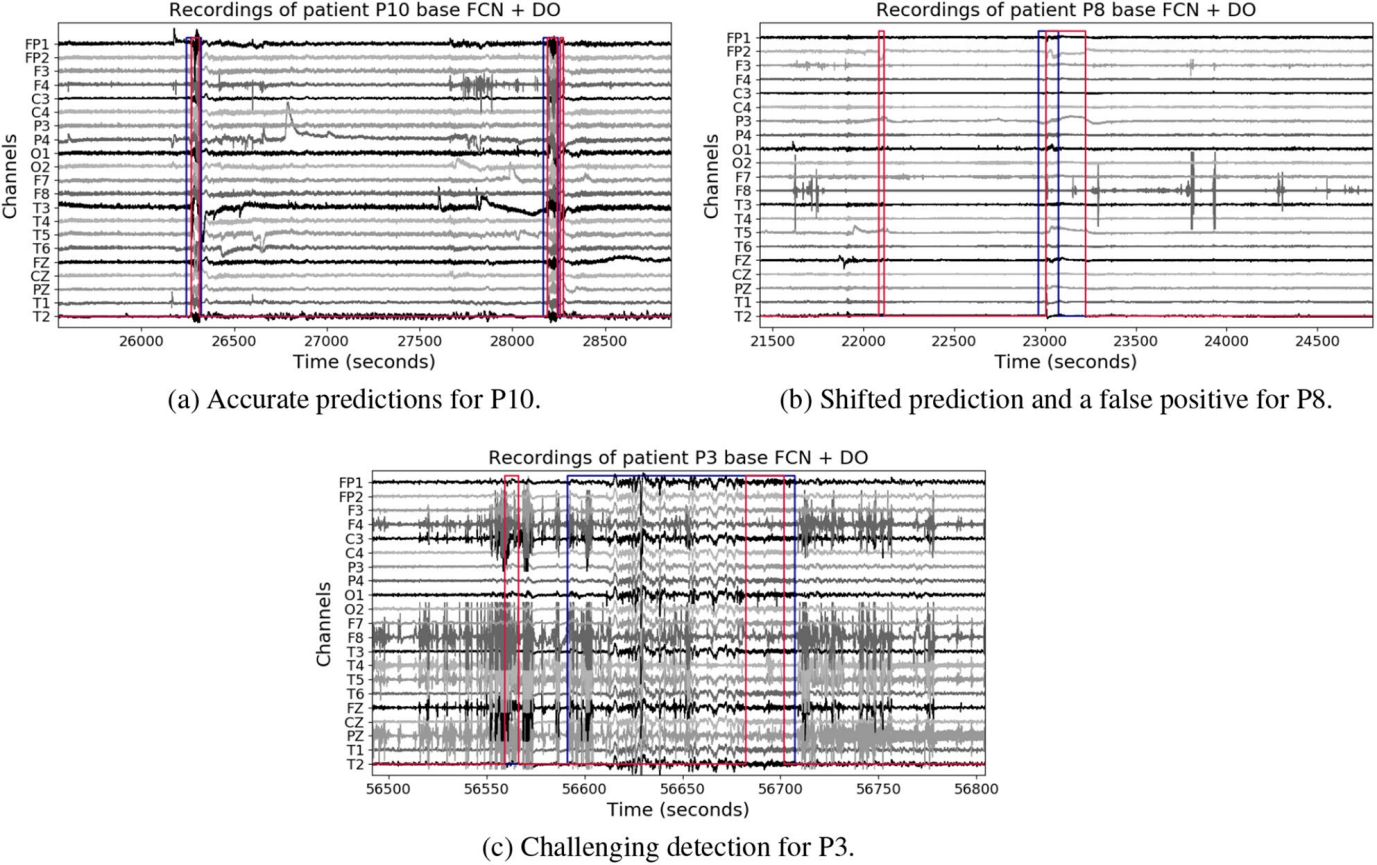
Qualitative results in the evaluation recordings of the best scalp model in the EPILEPSIAE patients. The y-axis shows the monopolar electrodes, and the x-axis the time in seconds. Blue lines correspond to annotations, and red lines to predictions.

For demonstrative purposes, we visualized the predictions of the models for three chosen patients, as shown in Fig. 7. Here, Fig. 7A corresponds to an example of two seizures that were correctly detected; Fig. 7B presents a false positive detection and a short seizure that is not well delimited; finally, Fig. 7C provides an example of a patient with noisy recordings. This kind of segments could explain a reduced ability for the model to identify seizures, and thus, the reduced overall performance.

#### Intracranial electrodes

**Figure 8:**
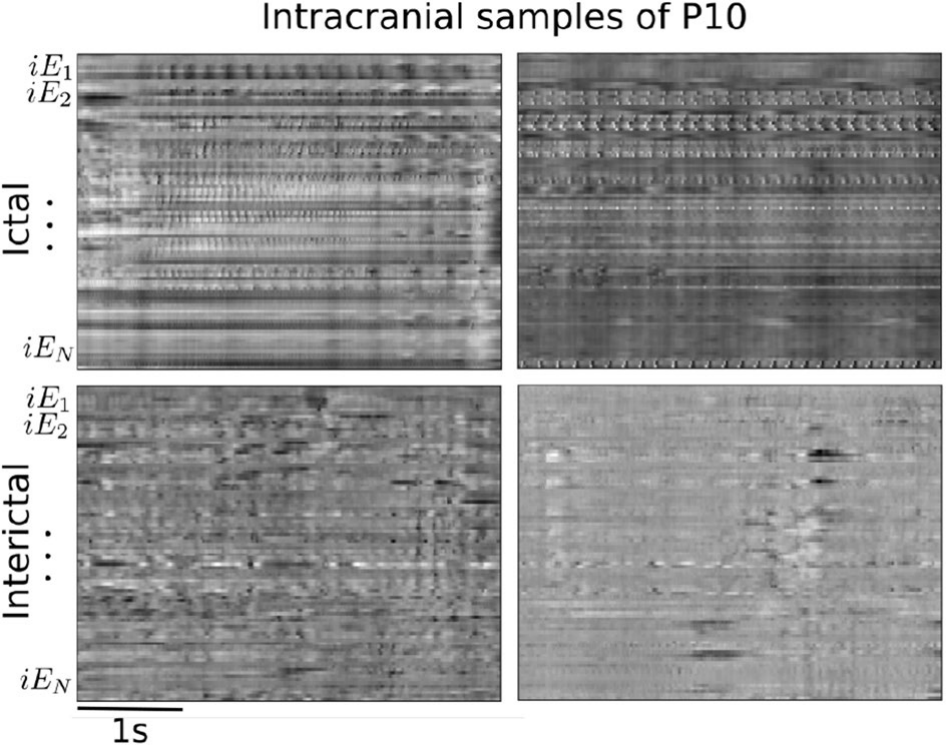
Signal representation of intracranial electrodes in monopolar configuration (57 intracranial contacts for P10). The y-axis corresponds to the intracranial channels labeled as $iE_N$ and the x-axis to the temporal dimension. Top and bottom rows present examples of ictal and interictal periods, respectively.

 With the objective to evaluate the contribution of invasive recordings to our seizure detection algorithm, we included the intracranial electrodes information in addition to the scalp ones. For this, we trained patient-specific models since the locations of implanted contacts were unique to each patient. We selected the patients with the highest number of seizures (at least 9), giving us four patients in total (P3, P6, P8 and P10). Examples of ictal and inter-ictal instances are shown in Fig. 8. To determine the best model for each patient, we explored different configurations between monopolar and bipolar intracranial and/or scalp electrodes as input. Besides, we initialized each patient-specific model with the best model found with scalp recordings from all the EPILEPSIAE patients as a transfer learning strategy.

**Figure 9:**
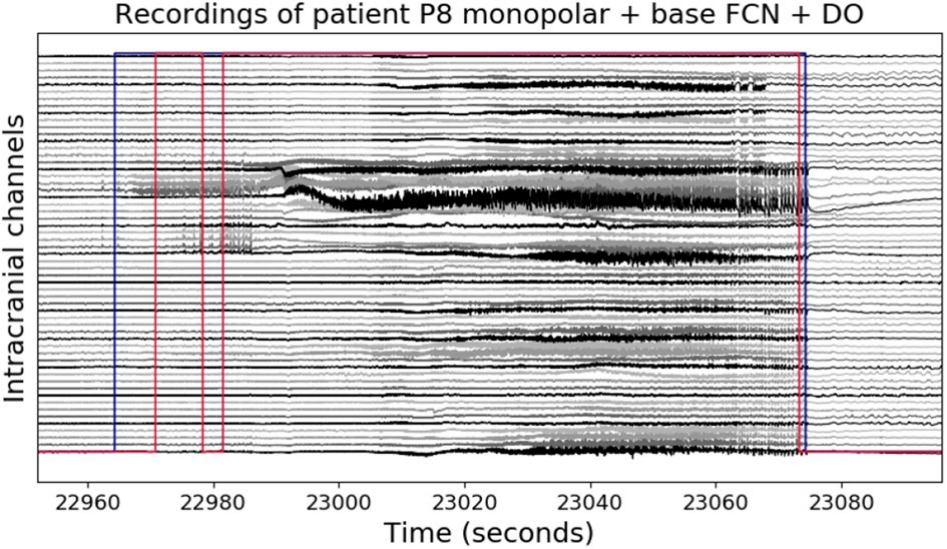
Detections in the evaluation recordings of P8 with the monopolar-intracranial-finetuning setup. The y-axis shows the intracranial electrodes in monopolar configuration, and the x-axis the time in seconds. Blue lines correspond to annotations and red lines to predictions.

The results with the best model obtained for each patient are summarized in Table 2 (for a complete comparison between experiments with intracranial data refer to Table 4in the supplementary material). Patient P3 is not displayed because neither model could learn to detect seizures in its recordings. For P8, we identified that training models only with intracranial electrodes (denoted as *intra*) improved all the metrics compared to the scalp-only models. For this patient, the results further improved when we finetuned the cross-patient monopolar scalp model to the patient-specific monopolar intracranial model (Monopolar-intra-FT in Table 2). We visualized one of these seizures in Fig. 9; here, it is possible to observe that our detection (red lines) is disrupted at the beginning of the seizure, when ictal patterns are not as evident as in the subsequent seconds, but the seizure end is accurately identified. For P10 and P6 patients, the combination of both electrode types further improved the results in the bipolar and monopolar configurations respectively, even though most of the metrics for P6 remained deficient. Consistently, patient-specific models trained from scratch only with scalp electrodes in a bipolar configuration (Bipolar-scalp-zero in Table 4 from the supplementary materia) reached similar results to those reported in the cross-patient model (Figure 1 in the Supplementary material) for P8 and P10.

**Table 2:** Evaluation metrics using the First Seizures configuration with intracranial data from three patients of the EPILEPSIAE database.

First seizures with intracranial electrodes							
Patient	Best model	Acc.	Spec.	Prec.	Rec.	F1	AP
P8	Monopolar intra FT	99.6	83.1	94.9	82.8	88.4	90.3
P10	Bipolar both zero	99.9	83.6	97.6	83.5	90.0	89.3
P6	Monopolar both zero	99.5	8.12	12.7	7.82	9.68	3.04
Mean ± std	–	$99.7 \pm 0.17$	$58.3\pm 35.5$	$68.4\pm 39.4$	$58.0\pm 35.5$	$62.7\pm 37.5$	$60.9\pm 40.9$

## Discussion

In this work, we have explored and validated state-of-the-art strategies based on deep learning approaches for the automatic detection of epileptic seizures. The main advantage of our method is that it does not need any estimation of pre-selected features for their execution, which usually requires the intervention of trained technicians or engineers, thus limiting their use to restricted environments. Besides, our method requires minimum pre-processing stages from raw EEG signal, i.e. signal saturation at $\pm \,250\,\upmu$V to reduce noise, avoiding filtering and domain transformations. Our algorithms tested in hundreds of hours of scalp and intracranial recordings evidenced high performances on the usual metrics of accuracy and sensitivity, which are comparable or even higher than those reported in previous works. In addition, due to the large class imbalance between ictal and inter-ictal episodes, we report more appropriate metrics such as the precision, the F-measure, the AP, and recall, for which we reached high results for some of the analyzed patients.

The comparison of our results with previous studies is not straightforward since there is not a standard evaluation for the seizure detection problem; each author reports its own metrics and the definition of a positive or negative sample depends on the window length being analyzed. To provide the possibility to fully reproduce our results and allow future comparisons, we make publicly available our source code and trained models in GitHub. Despite this, we still compared our method with the metrics of a recent report in the CHB-MIT dataset^32^. In this work, the authors trained and evaluated their algorithm for each patient individually, but they did not specify how data was split into train and test sets. Comparing the average accuracy and specificity of our best model in the First Seizures configuration ($99.3\pm 1.16$ and $99.6\pm 0.59$ respectively), we obtained a performance that is superior to the one reported by the authors in the inter-ictal vs. ictal task (accuracy of 62.3 and specificity of 63.3), but their recall (61.2) was greater by 3 points. However, as mentioned above, due to the large class imbalance of ictal to inter-ictal episodes, the accuracy and specificity might be presented in conjunction with more robust metrics to imbalance tasks, such as the precision or the FPR/h.

In another recent work using the same CHB-MIT Scalp EEG database^29^, the average recall or sensitivity was 85% for cross-patients models in a LOO configuration, but the authors do not report precision. Instead, they computed the false positive rate per hour (0.8/h) for their predictions over every 30-s segment of the signal. In our evaluation of false positives in 30-s windows, the average FPR/h of 7.81 is inflated by the low performance of 4 patients (6, 12, 14 and 16), which also affected the precision and recall metrics. Considering only the FPR/h values within the 95% percentile, our average rate 0.39/h is half the one reported in^29^. To compare the performance for each patient, the authors presented graphs with individual metrics as the difference of their performance with a previous approach^35^, making the comparison difficult. Despite this, we agreed on the performance of patients with the most challenging seizures to detect, such as 6, 12 and 16.

In order to improve the detection performance and to provide the model with better capabilities for generalization, we included prior information from the recordings in the First Seizures setup. Since the model has already seen seizure samples from all patients, all the average metrics increased. In particular, while the metrics for the patients identified as hard in the LOO setup improved, the performance of the easy patients remained almost unchanged. In addition, under the First Seizures configuration, the FPR/h was zero for 18 out of the 24 patients, meaning that there are no false positives in the predictions.

In the models with EPILEPIAE patients, we observed that finetuning from models that have already been trained to identify ictal from inter-ictal activity in a bipolar configuration of electrodes did not improve the results as expected. The metrics were similar when models were trained from scratch. Even so, our model achieved high accuracy and specificity values close to 100% ($98.0\pm 3.34$ and $98.3\pm 3.40$ respectively). However, the other metrics were considerably low, having average values over 25%. This disparity can be explained by the robustness of each metric to imbalanced data. When we included the intracranial channels, we found an improvement of seizure detection in two patients (P8 and P10), for whom the models with scalp data had satisfactory results. We also found a slight gain in performance for the other two patients, but overall, the algorithm failed to detect seizures in the recordings from these patients, as demonstrated in the experiment using only scalp electrodes. We attribute the improvement of including intracranial electrodes to the better spatial localization, and less artifacts than those present in scalp EEG^36^. In addition, seizure patterns may emerge stronger over specific electrodes according to the origin of the seizure activity. For a better validation of the contribution of intracranial electrodes to the seizure detection problem, we must test these configurations in a more representative group of patients.

In conclusion, the large variability in the evaluation metrics for different patients reflects the difficulty of the problem, and the challenges in defining a cross-patient model that can generalize well for patients that it has never seen before. Cross-patient models can be improved if the model learns from instances that include the first recorded seizures. Finally, the inclusion of intracranial channels in addition to the scalp ones in a patient-specific model improved all metrics for some patients, suggesting that both types of recordings carry complementary information that is needed for a better detection of epileptic seizures.

## Methods

### Dataset description

#### CHB-MIT scalp EEG database

 This epilepsy dataset consists of scalp EEG signals from 23 pediatric subjects with intractable seizures admitted at the Boston Children’s Hospital^3^, which is publicly available at PhysioNet’s website^28^. The recordings were acquired using the International 10-20 System for electrode positioning in bipolar configuration, sampled at 256 Hz with 16-bit depth. Each recording was annotated by experts indicating seizure beginning and ending times in seconds. From the 24 cases, we analyzed a total of 941.6 h of inter-ictal activity and 3 h of ictal acitivity from 181 annotated seizures (see Table 1 in the supplementary materia for a complete description of cases).

#### EPILEPSIAE

 The European Epilepsy Database contains high-quality EEG recordings of 275 epilepsy patients^37^. Raw EEG data comprise at least 5 days of continuous recording for each subject, with several scalp and/or intracranial channels. The dataset contains metadata information about recordings and patients, including the EEG onset and offset times for all of the seizures (in a date-hour format), the electrodes involved in the seizure activity (primary and secondary propagations), additional classification of the seizures, among others, all annotated by specialist neurologists. From the 275 patients, only 50 have intracranial recordings, and 225 have additional scalp EEG. Intracranial channels consisted of implanted grids, strips and/or depth electrodes, with a variable number of invasise contacts depending on each patient. Surface recordings consisted of 10-20 or 10-10 standard EEG electrode placement. From these patients, we selected the ones that fulfilled the requirement of simultaneously having intracranial and scalp recordings (n = 10 patients), the latter with the same channels of the CHB-MIT patients for comparison purposes. All signals were originally sampled at 1024 Hz with a 16-bit resolution and then downsampled to 256 Hz for the analyses. From this database, we analyzed a total of 1302.2 h of inter-ictal activity, and 2.5 h of ictal activity from 109 annotated seizures (see Table 1 in the Supplementary material for a complete description). Seizures segments shorter than 9s were discarded.

### Data representation

#### Signal representation

 To change from signals to images domain, we took the raw amplitude at each sample and channel as the pixel attribute of a 2D image, where the rows corresponded to the different EEG channels aligned vertically, and the columns to the temporal dimension. For the CHB-MIT dataset, we standardized the order of electrodes across all patients by choosing the 23 common bipolar channels among all the registers. We then split the time course of EEG signals into segments (windows) of 4-s of duration, so each resulting image had dimensions of $N \times (4\times SamplingRate)$, where *N* is the number of channels (recorded electrodes), and *SamplingRate* the number of samples per second depending on the dataset. We wanted to design seizure detection methods requiring a minimum of pre-processing steps, as these last require several subjective considerations that we wanted to avoid. For this and intending to reduce the more prominent artifacts, we saturated the scalp EEG signals with a threshold amplitude of $\pm \,250\,\upmu$V and used the raw amplitudes without any further pre-processing.

**Figure 10:**
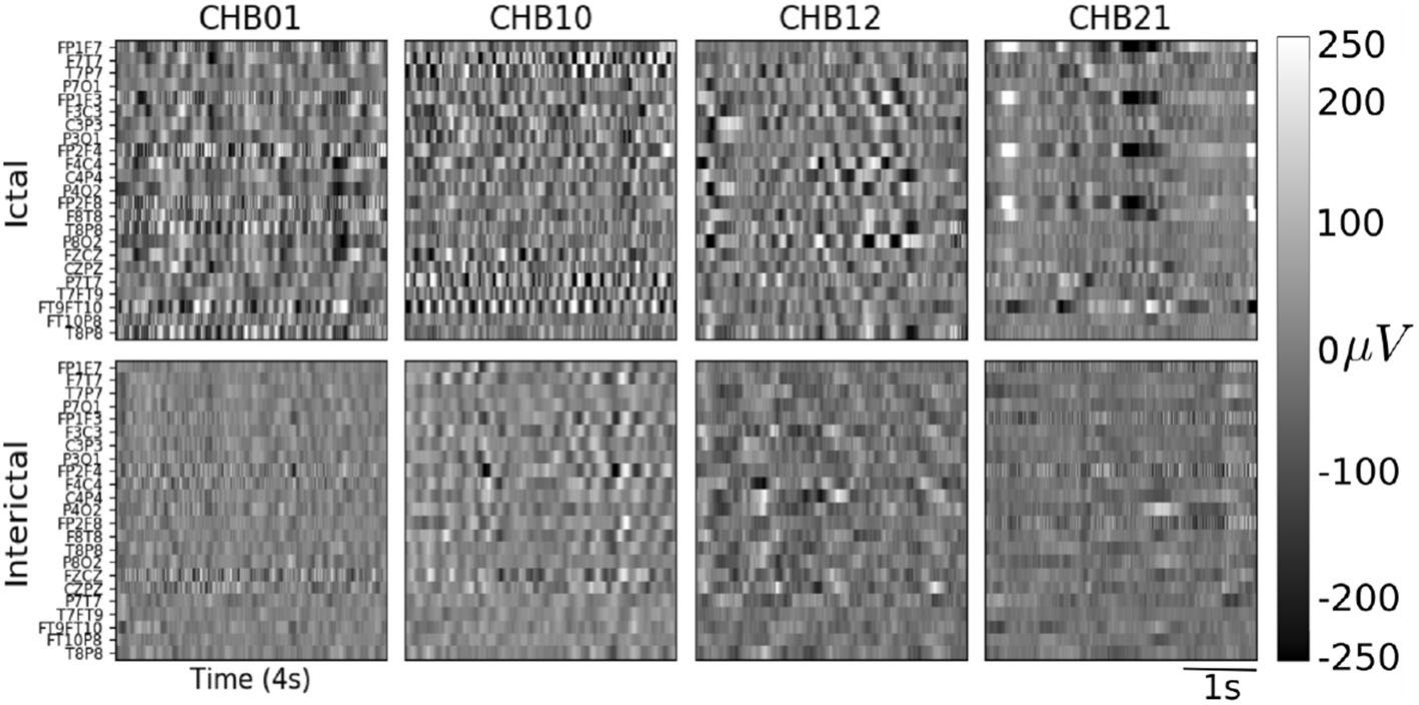
Examples of the signal representation taking the amplitude as the pixel attribute for four patients from the CHB-MIT Scalp dataset. The height encodes the channels in bipolar configuration, and the width the window length (4 s) with the aspect ratio adjusted for visualization purpose. The colorbar displays the amplitude range of the signals in $\upmu$V. Top row: ictal examples, bottom row: interictal examples.

Different examples of the obtained images for ictal and inter-ictal instances are shown in Fig. 10. In this figure, the amplitude of each time point is encoded through a grey scale. This visualization offers the posibility to determine changes in the spatial and temporal dimensions during an ictal and inter-ictal period, and to identify the electrodes that are mostly involved in a seizure.

##### Data augmentation

 Data augmentation is crucial to avoid overfitting in Neural Networks^38^. Previous studies^8,21^ have defined augmentation strategies to train CNN using additional examples defined by overlapping temporal windows with small shifts. Due to the spontaneous nature of epileptic seizures and their short duration compared to the extensive recording of inter-ictal activity, we augmented the number of training examples for the ictal class. To do so, we created overlapped images during the complete seizure duration with a time-shift between consecutive windows of 1/8 and 1/16 s for the CHB-MIT and EPILEPSIAE datasets, respectively. Even after data augmentation, the class imbalance remained. Thus, the inter-ictal instances were then randomly subsampled such that a fraction of them was close to the seizures, by defining a temporal closeness parameter as 150s before and after, and the other fraction was sampled far from the seizures. The inter-ictal instances close to the seizures were augmented with the same strategy mentioned before, but with a time-shift between consecutive windows of 1/4 and 1/8 s for the CHB-MIT and EPILEPSIAE datasets, respectively. No overalapping between consecutive windows was used for inter-ictal instances far from the seizures.

### Detection algorithm

#### Fully convolutional networks (FCN)

 Convolutional Neural Networks have been used to address object recognition tasks due to their ability to encode the input in multiple representations on its hidden layers, and propagate it through the network to produce an output with a final classification stage that is usually a fully connected layer. To make dense predictions for per-pixels tasks, CNN changed to FCN by replacing fully connected layers with convolutional layers, with kernels of the same dimension as the input^39^. The advantage of using FCNs is that they produce dense outputs from arbitrary-sized inputs, thus, they are able to process recordings of different lengths. For our classification problem, the inputs of the networks corresponded to the raw EEG matrix images, which were split in two classes: ictal and interictal (positive and negative classes respectively).

**Figure 11:**
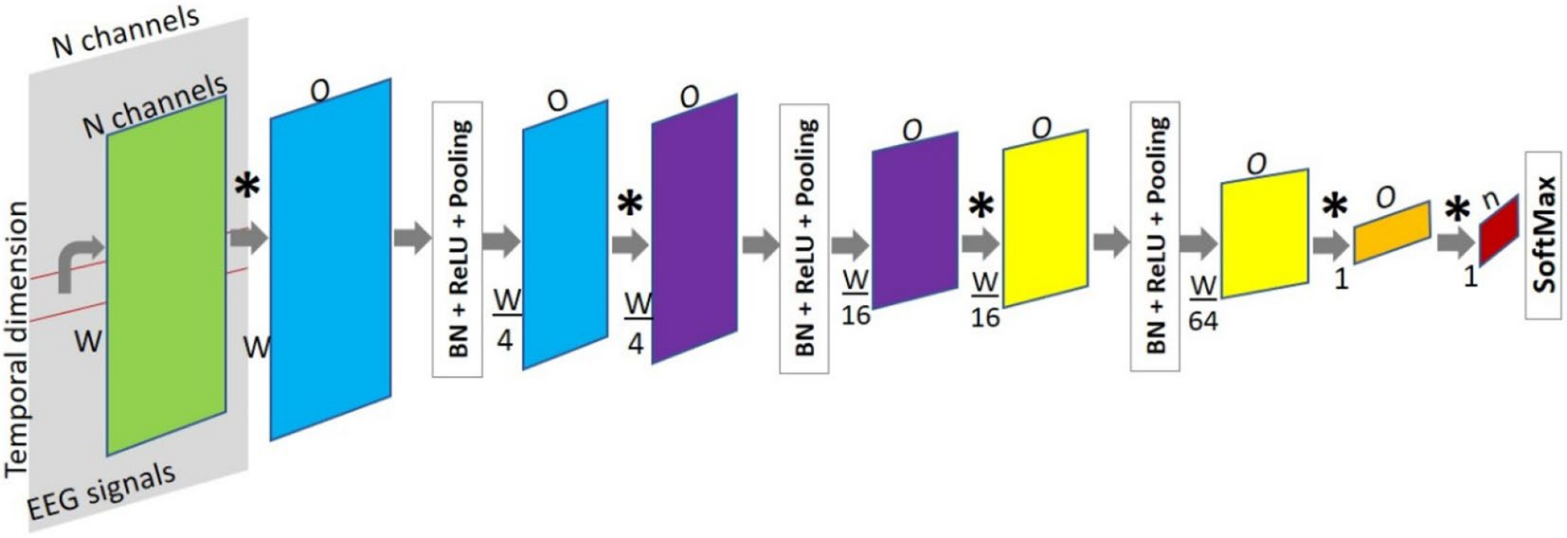
Proposed FCN architecture for the automated classification of EEG recordings into ictal and interictal states. The temporal dimension is reduced by 4$\times$ at every block (blue, purple and yellow). *A convolution operation, *n* is the number of classes, W is the window width, and *O* is the number of output channels in the respective convolutional layer.

For classifying neural activity states we designed a base FCN network with 3 blocks. Each block consisted of convolution, Batch Normalization, non-linear transformation ReLU, and Pooling layers, followed by two fully convolutional layers and a final SoftMax layer, as shown in Fig. 11. The purpose of the SoftMax layer was to rescale the elements from the output of the last convolutional layer in the range (0, 1), such that the final output of the network was a probability vector of either class.

Initially, the number of input channels for the convolutional layers corresponded to the same channels of the recordings, and then, the channel dimension denoted the number of features learned at each convolutional layer. The dimensionality of the input was reduced only along the temporal axis in each pooling layer. The kernels in the last convolutional layers, which replace the fully connected ones, were defined such that the network produced a class label every window of a fixed size. In this way, the input could be of variable length and the number of predictions depended on the length of the input (recording) to be evaluated.

We further introduced two regularization strategies for Deep Neural Networks. The first one, referred as Dropout (DO), prevents that the units of the model from co-adapting to the training data by randomly dropping out units and their connections^40^. The second one, *L2* regularization (weight decay) helps the network to generalize better.

Due to the limited number of patients, seizures and their short duration, we used a transfer learning strategy, called finetuning, to train a model in a database that does not have sufficient size^41^. In brief, once a model has been trained on a base dataset for a specific task, the learned features are transferred to a second network that is trained with samples from a different dataset for a new task. The weights of the pretrained model are said to be finetuned by continuing backpropagation. All the layers from the network can be finetuned, or only a fraction by freezing the weights of the layers that want to be preserved. This process will work if the learned features could be generalized, and if the nature of both the tasks and data is similar.

### Models and evaluation

#### CHB-MIT Scalp EEG

 In the CHB-MIT Scalp EEG dataset, we defined cross-patient models by splitting the recordings from the patients in two ways. First, in the 3 fold Cross-Validation (3FCV), we divided patients into three sets by training in two sets and evaluating in the remaining one, such that we covered the three possible combinations. Second, in the *Leave-One-Patient-Out* (LOO), 24 models were trained, each one using data from $k-1$ patients (where *k* is the total number of patients), and evaluated in all the recordings of the patient left out. In both configurations, we sampled 4s windows of the ictal and inter-ictal classes from the recordings of the patients in the train set, and evaluated our model in the complete recordings of the patients in the test set.

In addition, we followed an approach to train the models based on chronological information of the signals^42^, which we called First Seizures model. In particular, we defined a criterion to train the models with the initial 80% of the total seizures from each patient, and we evaluated in the remaining percentage of seizures and the associated inter-ictal recordings. Thus, the model can learn a representation from the information of the first seizures from all patients.

Based on the original architecture and the pooling operations over the temporal dimension, the model made predictions every 64 sample points.

#### Transfer learning in EPILEPSIAE

 For the EPILEPSIAE dataset, we evaluated a cross-patient model in the First Seizures setup for the scalp recordings. For the intracranial electrodes, we evaluated patient-specific models in the First Seizures setup.

To define the models for EPILEPSIAE patients using scalp electrodes, we departed from the parameter exploration conducted in the CHB-MIT dataset and used the base FCN architecture with DO and weight decay. First, we finetuned the best model in CHB-MIT patients to detect seizures on EPILEPSIAE patients. To do so, we adjusted the scalp electrodes in the same bipolar configuration, and initialize the weights of the new model with the pre-trained ones. The complete weights of the new model were optimized in the training partition for EPILEPSIAE patients. Alternatively, we trained models from scratch with ictal and inter-ictal examples from EPILEPSIAE patients, changing the electrode configuration (monopolar) and the number of filters in the convolutional layers.

To include the information from the intracranial electrodes, we trained patient-specific models in the first seizures model setup. The three strategies to train the models were: (i) training from scratch using only scalp or intracranial electrodes, and their combination, (ii) the models from (i) in a monopolar and bipolar configuration, and (iii) finetuning of a model pretrained in the first recordings with seizures of the 10 EPILEPSIAE patients, adapting the input size to the number of intracranial channels only. Given that the preexisting models were trained in scalp recordings, we included an initial convolutional layer that mapped from *N* intracranial channels to the number of scalp electrodes. The parameters for this new layer were learned during the training phase. The bipolar montage for the intracranial channels was defined differently for each patient as the difference between adjacent electrodes of the same electrode bundle, following the same strategy as in^43^.

#### Evaluation metrics

We evaluated the performance of the algorithms using the standard metrics of seizure detection problems^4^: accuracy ((true positives+true negatives)/(positives+negatives)), specificity (true negatives/(true negatives+false positives)) and sensitivity (true positives/(true positives+false negatives)), and also included those of detection problems: precision (true positives/(true positives+false positives)), recall or sensitivity, F-measure (harmonic mean of precision and recall), also called F1 score, and Average Precision (AP), which is defined as the area under the precision-recall (PR) curve built with different confidence thresholds according to the probability of belonging to the ictal class. The global performance of a model was given by the average ± standard deviation of the metrics across all patients in a given evaluation set.

## Supplementary Information

Supplementary Information.
