# Supplementary material for: Automatic seizure detection based on imaged-EEG signals through fully convolutional networks
Source: Sci Rep. 2020 Dec 11;10:21833. doi: 10.1038/s41598-020-78784-3 (PMC7732993; doi:10.1038/s41598-020-78784-3)
Supplement: Supplementary file 1 — Supplementary Information. [file 41598_2020_78784_MOESM1_ESM.pdf]

Automatic Seizure Detection based on imaged-EEG signals through Fully Convolutional Networks

Catalina Gomez[1], Pablo Arbelaez[1], Miguel Navarrete[2], Catalina Alvarado-Rojas[3], Michel Le Van Quyen[4], and Mario Valderrama[1,*]

[1]Universidad de los Andes, Department of Biomedical Engineering, Bogotá, Colombia
[2]Cardiff University, School of Psychology, Brain Research Imaging Centre, Cardiff, UK
[3]Pontificia Universidad Javeriana, Department of Electronic Engineering, Bogotá, Colombia
[4]Laboratoire d'Imagerie Biomédicale, INSERM, Paris, France
[*]mvalderr@uniandes.edu.co

ABSTRACT

Supplementary material

Statistics of CHB-MIT Scalp EEG and EPILEPSIAE patients.

Table 1 summarizes the patient statistics of the CHB-MIT Scalp EEG Database and the sample of patients from EPILEPSIAE.

Model Configuration

We explored different configurations and hyperparameters for the FCN architecture in the 3FCV scenario. The best strategy from these tests was used to train the LOO models. We started from the base model described in the Detection Algorithm section and evaluated the effect of changing:

- number of filters $nf \in \{64, 128, 256\}$
- kernel size $ks \in \{3, 51\}$
- learning rate schedule
- including dropout layers
- weight decay to the cost function
- input information: time and frequency domain

The results of experiments under the different configurations described above are shown in Table 2. Within experiments, each line corresponds to the test metrics of each fold, reported as the average and standard deviation over the test patients of the respective fold.

The effect of changing the number of filters mostly affects the performance of the patients evaluated at the third fold, improving the F-measure from 35.7 to 42.0. However, the average F-measure of the patients in the second fold decreased and presented a slight improvement in the first fold patients. We kept the base FCN number of filters. In the model with a larger kernel ($ks = 51$), the corresponding time in seconds was 200 ms, while the base model had $ks = 3 = 12ms$. We expected that having a larger kernel size and hence, more temporal information, would increase the performance considerably, but it required more parameters to be learned in the first convolutional layer with the same amount of training samples.

We include an adaptive learning rate to the base model, in which we adjusted the learning rate by a factor of 0.1 when the validation loss reached a plateau and stopped decreasing. This learning rate adjustment improved the performance for patients in the first and third fold. We also tested two strategies to reduce the overfitting of the model during the training phase: dropout layers (DO) and weight decay ($wd = 1e^{-4}$) to the error function. We defined DO layers in all convolutional layers but with different probabilities, 0.1 for the first layers because we did not want to lose first level features, and 0.3 for the two last convolutions. These regularization strategies allowed the model to perform better in the patients with harder seizures to detect

Table 1. General statistics of patients from CHB-MIT and EPILEPSIAE databases. No. channels in CHB-MIT patients corresponds to scalp electrodes in bipolar configuration, and in EPILEPSIAE to scalp electrodes in monopolar configuration + number of intracranial electrodes.

CHB-MIT Scalp EEG Database					
Patient	No. channels	No. seizures	Time seizures (s)	Avg. seizures length (s)	Time interictal (h)
chb01	23	7	442	63.14 ± 30.47	40.55
chb02	23	3	172	57.33 ± 41.86	35.27
chb03	23	7	402	57.43 ± 8.38	38.00
chb04	23	4	378	94.50 ± 30.88	156.1
chb05	23	5	558	111.6 ± 9.45	39.00
chb06	23	10	153	15.30 ± 2.87	66.73
chb07	23	3	325	108.3 ± 30.44	67.05
chb08	23	5	919	183.8 ± 49.19	20.01
chb09	23	4	276	69.0 ± 7.70	67.87
chb10	23	7	447	63.86 ± 17.24	50.02
chb11	23	3	806	268.7 ± 418.6	33.79
chb12	23	27	989	36.63 ± 19.0	20.69
chb13	23	10	440	44.0 ± 22.40	11.00
chb14	23	8	169	21.12 ± 8.68	26.00
chb15	23	20	1,992	99.60 ± 53.58	39.01
chb16	23	8	69	8.62 ± 2.50	17.00
chb17	23	3	293	97.67 ± 15.04	20.01
chb18	23	6	317	52.83 ± 14.43	34.63
chb19	23	3	236	78.67 ± 2.08	28.93
chb20	23	8	294	36.75 ± 6.25	27.60
chb21	23	4	199	49.75 ± 28.52	32.83
chb22	23	3	204	64.0 ± 8.72	31.00
chb23	23	7	424	60.57 ± 32.57	26.56
chb24	23	16	511	31.94 ± 18.41	12.00
Total	-	181	11,015	-	941.6
EPILEPSIAE					
P1	21 + 71	9	1,091	121.2 ± 22.91	162.6
P2	21 + 94	5	429	85.80 ± 28.21	37.97
P3	21 + 70	10	1,233	123.3 ± 26.80	124.5
P4	21 + 84	8	1,267	158.4 ± 70.56	48.05
P5	21 + 40	5	130	26.00 ± 4.243	13.86
P6	21 + 98	9	1,060	117.8 ± 15.34	177.4
P7	21 + 38	7	394	56.29 ± 41.78	162.6
P8	21 + 57	20	2,079	103.9 ± 36.73	117.9
P9	21 + 84	23	488	2061 ± 8933	344.4
P10	21 + 96	13	733	56.38 ± 35.43	110.9
Total	-	109	8,904	-	1,300.2

Table 2. Global averaged results (%) in all the records of the test patients for the variations of the base FCN in the 3FCV configuration. Each row within each experiment corresponds to one of the three folds (1-3). The best experiment is in bold.

3FCV				
Exp.	Precision	Recall	F-measure	AP
**Network architecture**				
Base FCN	40.0 ± 33.8	32.07 ± 26.8	34.9 ± 30.0	30.2 ± 33.0
$nf = 128$	61.3 ± 28.6	58.8 ± 25.3	59.9 ± 26.8	57.1 ± 30.6
	33.9 ± 27.6	56.7 ± 30.0	35.7 ± 23.8	27.9 ± 24.9
FCN	38.8 ± 30.0	33.4 ± 21.0	33.4 ± 25.8	28.5 ± 26.4
$nf = 64$	54.6 ± 24.4	51.7 ± 20.8	52.2 ± 21.9	46.9 ± 25.6
	30.2 ± 25.1	55.0 ± 31.3	30.6 ± 22.3	21.8 ± 20.3
FCN	40.4 ± 35.3	35.5 ± 25.6	36.5 ± 31.0	31.6 ± 32.4
$nf = 256$	58.6 ± 29.1	57.6 ± 24.8	57.6 ± 27.2	55.0 ± 31.7
	39.0 ± 24.1	58.2 ± 31.1	42.0 ± 22.3	33.5 ± 22.7
FCN	40.6 ± 36.0	32.6 ± 28.2	33.1 ± 31.0	28.8 ± 33.0
$nf = 128$	55.9 ± 29.8	48.3 ± 21.6	51.2 ± 24.9	48.9 ± 30.1
$ks = 51$	35.5 ± 27.3	52.4 ± 29.6	34.6 ± 22.6	26.0 ± 22.4
**Training curriculum**				
Base FCN	44.9 ± 38.0	57.8 ± 27.6	40.0 ± 33.6	36.2 ± 33.6
LR plateau	60.7 ± 23.4	46.8 ± 21.2	51.5 ± 21.3	47.3 ± 23.6
	36.8 ± 22.5	53.9 ± 27.1	39.5 ± 23.6	30.3 ± 20.6
Dropout	36.8 ± 33.2	45.2 ± 33.0	34.5 ± 30.5	32.1 ± 33.3
0.1- 0.3	64.7 ± 26.2	47.1 ± 20.9	53.5 ± 23.3	50.8 ± 25.6
	51.2 ± 35.3	44.7 ± 30.2	46.0 ± 32.9	43.2 ± 36.2
**Dropout**	45.0 ± 36.8	35.4 ± 25.7	35.6 ± 30.7	33.6 ± 33.5
**0.1- 0.3**	64.3 ± 26.4	55.9 ± 19.2	58.6 ± 24.0	56.9 ± 26.2
**wd**	51.2 ± 36.0	45.5 ± 29.3	46.6 ± 33.0	43.4 ± 36.3
**+ Frequency information**				
Base FCN	37.4 ± 29.6	32.9 ± 24.1	32.8 ± 27.8	30.5 ± 30.7
+ Filters	63.0 ± 29.9	58.8 ± 24.7	60.4 ± 27.3	58.5 ± 31.3
	20.3 ± 20.5	51.7 ± 26.5	24.6 ± 23.9	16.6 ± 18.1

(third fold), improving the F-measure in more than 10 points compared to the base model, while keeping the performance of the other folds almost constant.

To include frequency information, we filter the signals at different bands (0-7,7-14 and 14-49 Hz)[?], and add them to the original 23 channels, thus having a total of 92 channels. Then, we generate the ictal and interictal instances using the strategy described in the *Signal Representation* section, and trained a model with the base FCN architecture. The results are shown in the bottom block of Table 2. We did not observe an improvement compared to the base model, and having a larger input required more trainable parameters.

Results on CHB-MIT patients

We summarize the individual performance of CHB-MIT patients under different model configurations in Table 3.

Experiments on EPILEPSIAE patients

A complete comparison of the experiments in EPILEPSIAE patients with scalp recordings is presented in Figure 1.

The results of the different strategies to include intracranial electrodes for the four patients from EPILEPSIAE are shown in Table 4, in which each row corresponds to a different experimental setup (best one in bold).

Table 3. Complete evaluation metrics for all patients in the CHB-MIT Scalp EEG database.

	3FCV						LOO						First Seizures					
Patient	Acc.	Spec.	Sens.	Prec.	F1	AP	Acc.	Spec.	Sens.	Prec.	F1	AP	Acc.	Spec.	Sens.	Prec.	F1	AP
chb01	99.93	83.08	83.03	91.81	87.2	92.58	99.87	99.96	69.63	83.23	75.82	81.38	99.9	99.98	67.4	86.88	75.91	77.5
chb02	99.92	60.08	60.03	75.78	66.99	69.97	99.97	100.0	80.23	95.67	87.27	90.08	100.0	100.0	72.22	92.86	81.25	78.31
chb03	99.79	64.53	64.43	63.87	64.15	61.06	99.30	99.41	62.19	23.68	34.30	17.64	99.66	99.8	83.55	76.97	80.12	83.76
chb04	99.88	10.44	10.38	10.36	10.37	3.67	99.93	99.98	19.44	46.23	27.37	21.95	99.92	99.96	22.13	20.58	21.33	14.29
chb05	99.89	79.16	79.08	92.07	85.08	87.54	99.86	99.98	69.31	92.36	79.19	81.65	99.96	99.99	79.27	90.49	84.51	85.67
chb06	99.59	17.86	17.81	3.03	5.18	1.30	98.69	98.74	12.58	0.63	1.20	0.37	99.95	99.97	38.39	35.25	36.75	27.1
chb07	99.95	74.88	74.85	87.74	80.78	83.20	99.92	99.96	68.31	69.11	68.70	71.51	99.93	99.99	67.66	90.63	77.48	72.16
chb08	98.8	24.56	23.59	57.72	33.49	27.84	98.44	99.48	17.98	31.09	22.79	18.00	99.11	99.6	20.64	24.01	22.2	14.12
chb09	99.99	89.14	89.13	98.70	93.67	93.58	99.98	100.0	84.51	98.00	90.76	93.49	99.99	100.0	79.03	100.0	88.29	84.02
chb10	99.92	72.78	72.71	94.75	82.28	82.32	99.94	99.99	82.05	93.74	87.50	90.25	99.71	99.92	79.2	90.6	84.52	89.52
chb11	99.72	72.18	71.99	84.06	77.56	83.97	99.92	99.96	93.08	94.55	93.81	95.82	94.73	97.94	85.74	93.71	89.55	94.22
chb12	89.50	20.18	19.24	2.64	4.64	2.06	49.49	49.35	59.3	1.55	3.03	1.41	98.31	98.99	56.97	48.06	52.14	37.67
chb13	98.46	10.04	9.03	15.98	11.54	3.45	98.18	99.17	10.40	12.31	11.28	5.91	97.77	98.91	59.33	62.08	60.67	67.87
chb14	99.69	1.21	1.04	1.45	1.21	0.12	0.22	0.04	100.0	0.18	0.36	0.11	99.67	99.73	17.76	4.80	7.56	1.58
chb15	98.10	16.84	15.65	24.14	18.99	11.56	95.43	96.26	37.65	12.66	18.95	7.81	97.63	97.93	85.13	49.43	62.54	39.78
chb16	89.05	42.81	42.75	0.44	0.87	0.26	94.16	94.22	33.7	0.65	1.28	0.46	98.93	99.57	25.0	33.7	28.7	18.58
chb17	99.63	39.92	39.68	56.36	46.57	44.99	99.61	99.87	36.52	52.45	43.06	39.18	99.72	99.82	28.13	17.62	21.66	13.15
chb18	99.81	53.82	53.71	66.77	59.53	50.97	99.75	99.89	44.95	51.30	47.92	42.45	99.3	99.84	31.14	60.94	41.22	29.46
chb19	99.90	61.42	61.33	92.94	73.9	74.28	99.88	99.96	66.74	78.07	71.96	79.40	99.43	99.89	81.17	94.6	87.38	91.25
chb20	99.71	31.16	30.95	50.77	38.46	31.86	99.73	99.93	33.42	59.19	42.72	34.58	99.86	99.94	43.58	50.0	46.57	26.07
chb21	99.72	23.24	23.12	20.74	21.87	13.57	99.78	99.9	27.76	31.62	29.57	16.97	99.93	99.94	64.58	21.99	32.8	18.22
chb22	99.91	74.31	74.27	75.84	75.05	72.67	99.91	99.96	70.22	76.2	73.09	76.70	99.92	99.99	67.71	96.06	79.43	80.08
chb23	99.44	46.35	46.11	39.14	42.34	36.22	99.54	99.78	46.23	47.78	46.99	41.75	99.82	99.93	66.19	75.03	70.33	73.89
chb24	99.08	31.83	31.02	77.22	44.26	41.82	99.25	99.86	48.14	80.46	60.24	56.13	99.57	99.87	78.48	89.77	83.75	90.34

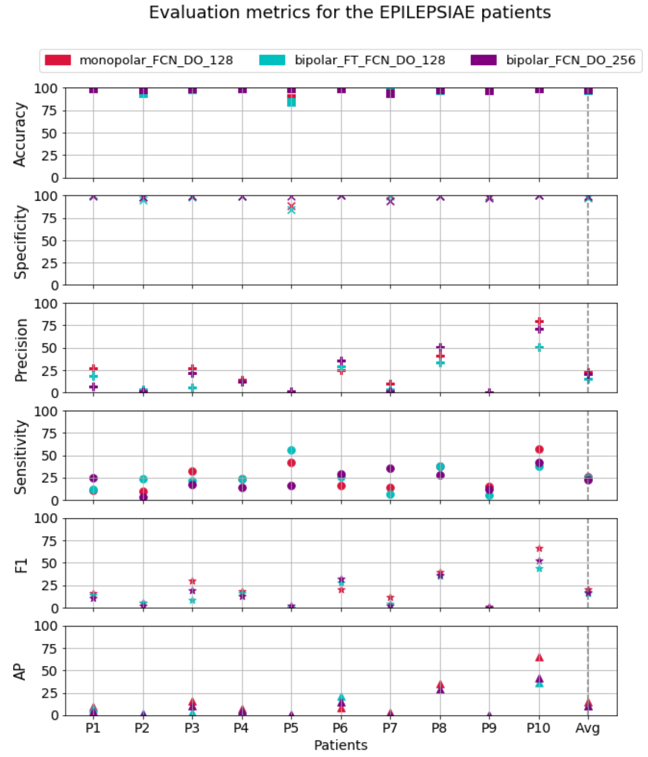

Figure 1. Comparison of experiments for the sample of EPILEPSIAE patients using scalp electrodes only. Each point in the x-axis corresponds to one patient and the average metric (Avg), and the y-axis to each metric. The colors and patterns encode a different experiment and metric, respectively.

Table 4. Evaluation metrics of first seizures model in four EPILEPSIAE patients using intracranial channels. The experiment name refers to the electrodes configuration, type and model initialization, respectively.

**First seizures model**								
	P8		P10		P3		P6	
Experiment	F1	AP	F1	AP	F1	AP	F1	AP
Monopolar intra zero	66.3	67.7	72.1	77.3	0.45	0.16	0.63	0.21
Monopolar scalp zero	51.7	48.5	64.4	64.0	0.46	0.19	0.63	0.17
Monopolar both zero	63.3	64.5	79.3	86.2	0.45	0.16	**9.68**	**3.04**
Bipolar intra zero	64.1	64.9	70.6	79.3	0.49	0.19	5.51	1.39
Bipolar scalp zero	23.6	15.3	52.9	53.4	0.45	0.15	0.63	0.17
Bipolar both zero	53.9	52.3	**90.0**	**89.3**	0.52	0.17	2.62	1.48
Monopolar intra FT	**88.4**	**90.3**	61.1	68.6	0.45	0.17	3.25	0.53
